# Investigating the Relationship Human Parvovirus B19 Infection in Benign and Malignant Salivary Gland Tumors 

**DOI:** 10.30476/dentjods.2025.102008.2330

**Published:** 2026-03-01

**Authors:** Ali Farhadi, Ali Dehghani Nazhvani, Fatemeh Faghihi, Mohammad Hadi Khademi, Hasan Rezazadeh

**Affiliations:** 1 Dept. of Medical Laboratory Sciences, School of Paramedical, Shiraz University of Medical Sciences, Shiraz, Iran.; 2 Dept. of Oral and Maxillofacial Pathology, Biomaterials Research Center, School of Dentistry, Shiraz University of Medical Sciences, Shiraz, Iran.; 3 Student Research Committee, School of Dentistry, Shiraz University of Medical Sciences, Shiraz, Iran.

**Keywords:** Salivary gland tumor, Parvovirus B19, Pleomorphic adenoma, Mucoepidermoid carcinoma, Adenoid cystic carcinoma

## Abstract

**Background:**

Parvovirus B19 is a common virus that can affect everyone, especially immunocompromised patients. The virus is present in various leukemias and solid tumors, and several B19-related diseases including autoimmune disorders, hepatitis, vasculitis, acute and chronic inflammatory diseases, and some thyroid cancers have been described.

**Purpose:**

This investigation aimed to quantify the frequency of human Parvovirus-B19 infection in salivary gland tumors, both benign and malignant.

**Materials and Method:**

In this cross-sectional study, 71 formalin-fixed, paraffin-embedded tissue specimens associated with benign and malignant salivary gland tumors, along with tissues from 30 normal salivary glands from the maxillofacial pathology laboratory of Shiraz Dental School, Chamran, and Rajai teaching hospitals (the main two major referral centers), were examined using nested-PCR to determine if B19 DNA was present. All data, including age, sex, location, and the presence of the virus, were considered, presented in tables, and statistically analyzed. The prevalence of B19 was compared to the normal salivary gland group.

**Results:**

B19 DNA was identified in 11 specimens (15.5%) out of the 71 available specimens from the patient group. None of the specimens from the normal salivary gland group tested positive for B19 DNA. Consequently, the prevalence of B19 in the patient group was significantly higher than that in the normal salivary gland group (*p* Value = 0.031). There was no significant relationship between age, sex, location, type of tumor, and the presence of B19 infection.

**Conclusion:**

Our results indicated a relationship between the presence of B19 parvovirus in the patient group compared to that in the control groups. Based on our study's findings, it can be assumed that B19 virus is likely to be presenting in both benign and malignant salivary gland tumors.

## Introduction

It has been stated that salivary gland tumors account for 0.3-0.5% of all malignancies and 6% of head and neck cancers [ 1 ]. The rarity and histological diversity within each tumor pose challenges for their study, leading to limited reviews in the literature. Most salivary gland tumors are benign (3.78%), with a higher prevalence in women (female-to-male ratio of 1.6 to 1) [ 2 ]. The parotid gland is the most often involved location (68.5%), and the age of affected individuals ranges from 1 to 88 years, with a mean age of 25 years [ 2 ]. The most common benign salivary gland tumor is pleomorphic adenoma, predominantly occurring in the parotid [ 3 ]. The smaller salivary glands are more likely to develop malignant tumors such adenoid cystic carcinoma and mucoepidermoid carcinoma [ 4 ].

The risk of salivary gland tumors may increase with factors such as smoking, radiation therapy, viral infection, certain nutritional deficiencies, and genetic mutations [ 5 ]. Evidence on salivary gland tumors indicates the presence of various viruses, including *Epstein-Barr* virus and cytomegalovirus, in tumoral tissues [ 6 ]. Investigations into the role of *human B19 virus* in various cancers including acute and chronic leukemia, thyroid cancers, and respiratory cancers have demonstrated higher virus levels in cancerous tissues compared to surrounding healthy tissues [ 7 - 9 ]. B19 virus belongs to the parvoviridae family, a single-stranded DNA virus [ 10 - 11 ]. The virus causes self-restricted diseases such as fifth disease or erythema infectiosum in non-immunodeficient individuals [ 11 - 12 ]. Additionally, the virus has been confirmed in various other diseases. Some researchers have established its role in various cancers such as leukemia and thyroid carcinoma [ 13 ]. However, the relationship between the virus and certain solid tumors, especially salivary gland tumors, has not been extensively studied [ 8 ]. Therefore, the purpose of this study was to look into the prevalence of *human Parvovirus-B19* in both benign and malignant tumors of the salivary glands.

## Materials and Method

### Sample Collection

The formalin-fixed, paraffin-embedded (FFPE) tissue samples from benign and malignant salivary gland tumors, as well as normal salivary glands, were all included in this cross-sectional investigation during the course of the previous five years. The samples were collected from the archives of Chamran and Rajaei hospitals and the Oral and Maxillofacial Pathology Department, School of Dentistry, Shiraz University of Medical Sciences, Shiraz, Iran. The study adhered to the relevant principles of the *Helsinki Declaration* and received approval from the Ethics Committee of Shiraz University of Medical Sciences, Shiraz, Iran (IR.SUMS.DENTAL.REC.1398.045). Every participant provided written, informed consent.

### Sample Preparation and DNA Extraction

Eight slices, each measuring 10 µm in thickness, were divided and collected for each case in microcentrifuge tubes. The FavorPrep™ FFPE Tissue DNA Extraction Micro Kit (Favorgen, Taiwan) was applied to the FFPE tissue slices in order to extract genomic DNA. Following extraction, the DNA was eluted using elution buffer and kept at -80°C. The total DNA amount was measured using a Nanodrop (ND- 1000) spectrophotometer (peQLab Biotechnology, Erlangen, Germany). According to Saiki *et al*. [ 14 ], each sample underwent a polymerase chain reaction (PCR) amplification to assess the quality of the extracted DNA using β-globin gene-specific primers PC03 and PC04, producing a 110 bp PCR result. Subsequent studies were limited to samples that tested positive for β-globin.

### Detection of Parvovirus B19

To increase the nested PCR method's sensitivity and specificity for amplifying parvovirus B19-DNA, a two-step process was used. Two sets of primers (Table 1) consisted of primers 4A and 4B as outer primers, generating 340 bp amplicons from the virus genome VP1 and NS1 gene region, and primers 4C and 4D as internal primers, producing 106 bp PCR products. All primers were custom synthesized by Bioneer (Daejeon, Korea). Twenty microliters of reaction mixture were used for the first round of nested PCR. It contained 500ng of extracted DNA, 13μL of Master Mix Red 2x (Ampliqon, Odense, Denmark), and 0.7 μM of each of the 4 A forward and 4 B reverse outer primers. For the nested PCR's second round, the reaction mixture composition was the same, but 1 μl of the first reaction result and 0.8 μM of the 4 C forward and 4 D reverse inner primers were added. The thermal cycling for reaction mixtures containing the outer primer set consisted of one cycle at 94°C for three minutes, 35 cycles at 94°C for one minute, 54°C for 45 seconds, and 72°C for 45 seconds, and a final cycle at 72°C for five minutes. Reaction mixtures for nested PCR products were subjected to 40 cycles of 94°C for 45 s, 56°C for 45 s, 72°C for 45 s, and a final cycle of 72°C for five minutes. Using a UV transilluminator (Kiatajhiz Co. Iran), SYBR Safe DNA gel dye was used to detect the 106-bp nested-PCR amplicons on 1.5% agarose electrophoresis gels (Thermo Fisher Scientific, USA). To ensure reliability and consistency, a negative control for every response was sterile distilled water, and positive specimens were confirmed by repeating the amplification twice.

**Table 1:** Sequence of used primers in the nested-PCR test

Product size	Sequences (5' - 3')	Primer name
340 bp	AACGCCTCAGAAAAATACCC	4A Forward outer
	TAAGTGCTGAAACTCTAAWGG	4B Reverse outer
106 bp	CAAAAGCATGTGGAGTGAGG	4C Forward inner
	CACYTTATAATGGTGCTCTGG	4D Reverse inner

Key: W=A, T, Y=C, T; PCR: polymerase chain reaction

### Statistical Analysis

SPSS version 25.0 (SPSS Institute) was used to conduct the statistical analysis. By using two-sided chi-square or, if necessary, Fisher exact tests, the relationships between the presence of viral agents and the clinicopathological features and/or the expression of cellular biomarkers were examined. The McNemar test was used to determine the significance of the variation in viral DNA prevalence between tumor tissue specimens and comparable peritumoral tissues. A *p* Value < 0.05 was considered statistically significant.

## Results

In this cross-sectional study, 71 paraffin blocks of benign and malignant salivary gland tumors and 30 paraffin blocks of normal salivary glands were examined. Twenty-six cases (36.6%) were male, and 45 cases (63.4%) were female, with a mean age of 50. Also, 12 males (40%) and 18 females (60%) were present in the group of normal salivary glands. Among the 71 studied cases, there were 25 (35.2%) pleomorphic adenomas, 22 (30.9%) mucoepidermoid carcinomas, 20 (28.1%) cystic adenoid carcinomas, 2 (2.8%) malignant salivary glands, 1 (1.4%) canalicular adenoma, and 1 (1.4%) low -grade pleomorphic adenocarcinoma. Normal salivary gland cases were obtained from mucocele lesions. Evaluation of site involvement showed that 50.7% of the tumors (36 cases) originated in minor salivary glands, and 49.3% (35 cases) existed in major salivary glands. Normal salivary gland cases were also from minor salivary glands located in the oral submucosa.

**Figure 1:**
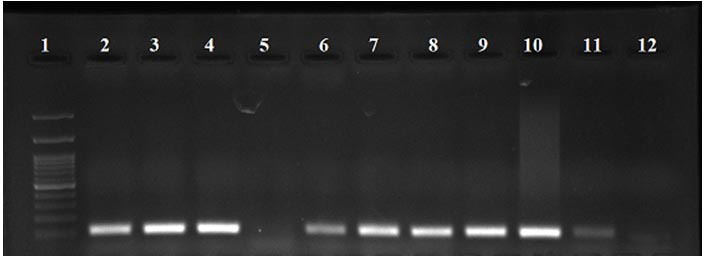
Electrophoreses of agarose gel for detection of B19-DNA. Lane 1: 100 bp DNA ladder, lane 2: Positive control containing 106-bp B19 virus DNA, lanes 3-11: Positive samples containing 106-bp B19 Virus DNA. lane 5: A negative sample, lane 12: Negative control;

B19-DNA was detected in 11 (15.4%) of the 71 cases (Figure 1). Among the B19 DNA positive cases, 6 cases (24%) were pleomorphic adenomas, 3 cases (13.6%) were mucoepidermoid carcinomas, and 2 cases (10%) were adenoid cystic carcinomas. All 30 normal salivary gland specimens tested negative for the B19 virus. Comparison of the presence of the B19 virus in the tumor group and normal salivary glands using the statistical test 'Pearson’s chi-Squared test (χ²)' revealed a significantly higher level of B19 virus DNA in the tumor group compared to the normal group (*p*= 0.031), but there was no relationship between the type of tumor and the presence of *human Parvovirus-B19* (*p*= 0.728). Additionally, using Pearson’s chi-squared test, there was no significant relationship between patient age (*p*= 0.527) and gender (*p*=1.000) with the presence of the B19 virus. The same holds true for the location of the tumor and parvovirus positivity (*p*= 0.343). Overall, the study's findings demonstrated that the patient group's level of *human Parvovirus-B19* was noticeably higher than that of the group with normal salivary glands. Nevertheless, no statistically significant correlations were found with any of the variables, including age, gender, tumor site, and type. 

## Discussion

Investigating the prevalence of *human Parvovirus-B19* in benign and malignant salivary gland tumors provides critical insights into potential viral associations; such knowledge may pave the way for enhanced diagnostic and therapeutic strategies. The treatment of salivary gland tumors is unsatisfactory due to the difficulty of completely removing the tumor and the poor response to chemotherapy and radiotherapy [ 15 ]. Therefore, understanding the pathogenesis can be of great help in developing new approaches to improve survival and the patient's quality of life.

After an initial B19V infection, viral DNA can persist in a number of human tissues, such as the tonsils, salivary glands, and thyroid [ 16 ]. A study on B19 infection revealed that this virus mainly affects children, and almost 50% of people encounter it until puberty. After the initial infection, the virus DNA remains permanently in some tissues for the entire lifetime, such as the bone marrow, tonsils, kidneys, muscles, thyroid, salivary glands, skin, liver, and heart [ 17 ]. Other studies have indicated B19's carcinogenicity in a high percentage of leukemia, thyroid tumors, and diseases with immune origins. For instance, a study on bone marrow samples of 249 patients with different types of leukemia confirmed the presence of the virus in all types of leukemia [ 18 ]. Another study investigating the presence of B19 in acute lymphoblastic leukemia in children found its presence in almost 47.5% of patients and 20% of the control group [ 19 ]. Similarly, a study on children with acute myeloblastic leukemia revealed the presence of the virus DNA, IgG, and IgM. Research on thyroid diseases demonstrated that 88% of patients with papillary thyroid carcinoma, 100% of patients with anaplastic thyroid carcinoma, and 100% of patients with Hashimoto’s thyroiditis had B19 virus capsid proteins, while there was either no virus in normal thyroid tissue or the percentage was very low [ 20 ]. Another study found parvovirus DNA in 86% of patients with thyroid tumors [ 21 ]. Additionally, in a study focused on papillary thyroid carcinoma, the amount of virus DNA in tumoral tissue was higher than in surrounding normal tissue [ 22 ].

To date, no study has been conducted on the prevalence of B19 virus in benign and malignant tumors of salivary glands. Therefore, the potential relationship between viral infection and the development of these tumors needs to be determined. In the recent study, 71 paraffin blocks of benign and malignant salivary gland tumors and 30 paraffin blocks of normal salivary glands were investigated. The samples included 36.6% benign tumors and 64.4% malignant tumors, with 63.4% women and 36.6% men. By performing nested-PCR, 15.5% of patients were positive for B19 virus DNA, and all normal salivary gland samples were negative, which was statistically significant. A study on nasopharyngeal angiofibroma, oral and oropharyngeal squamous cell carcinomas, and normal adjacent tissue showed that all nasopharyngeal angiofibroma samples were negative for the presence of parvovirus, and only one case had polyomavirus DNA. From ten samples of oral and oropharyngeal squamous cell carcinoma, seven samples showed B19 DNA, with four cases present in both tumoral tissue and surrounding normal tissue, and three cases only in normal tissue [ 23 ]. The lack of a significant relationship between the presence of the B19 virus and the development of oral and oropharyngeal squamous cell carcinoma may be due to inadequate samples and the lack of necessary tests to confirm viral gene expression and virus duplication in these tissues. However, the results suggest a possible role of the B19 virus in the pathogenesis of salivary gland tumors. In addition we recommend that further studies be conducted on the role of the B19 virus in salivary gland tumors, utilizing larger sample sizes and encompassing a broader spectrum of tumor types. Additional tests, such as immunohistochemistry and western blotting, should be employed to validate viral gene expression and demonstrate the duplication and replication of the virus in target tissues. These tests are essential to establish the role of the B19 virus in salivary gland tumors. Moreover, a thorough investigation into the specific role of B19 viral proteins in the development and spread of both benign and malignant salivary gland tumor cell lines is warranted. 

## Conclusion

Our results indicated a relationship between the presence of B19 parvovirus in the patient group compared to the control group. Considering the data from our study, it can be concluded that B19 virus is likely to persist in both benign and malignant salivary gland tumors.
